# Event-related potentials (ERPs) and hemodynamic (functional near-infrared spectroscopy, fNIRS) as measures of schizophrenia deficits in emotional behavior

**DOI:** 10.3389/fpsyg.2015.01686

**Published:** 2015-11-03

**Authors:** Michela Balconi, Simone Tirelli, Alessandra Frezza

**Affiliations:** ^1^Research Unit in Affective and Social Neuroscience, Catholic University of the Sacred HeartMilan, Italy; ^2^Department of Psychology, Catholic University of the Sacred HeartMilan, Italy; ^3^Sapienza University of RomeRome, Italy

**Keywords:** schizophrenia, NIRS, emotion, ERPs, integration

## Abstract

Recent research evidences supported the significant role of multimethodological neuroscientific approach for the diagnosis and the rehabilitative intervention in schizophrenia. Indeed both electrophysiological and neuroimaging measures in integration each other appear able to furnish a deep overview of the cognitive and affective behavior in schizophrenia patients (SPs). The aim of the present review is focused on the emotional dysfunctional response taking into account the multimeasures for emotional behavior, i.e., the event-related potentials (ERPs) and the hemodynamic profile functional near-infrared spectroscopy (fNIRS). These measures may be considered as predictive measures of the SPs’ deficits in emotional behavior. The integration between ERP and fNIRS may support both the prefrontal cortical localization anomaly and the attentional bias toward some specific emotional conditions (mainly negative).

## Emotional Impairment in Schizophrenia

Affective deficits are considered to be a core feature of schizophrenia (Kring and Elis, 2013). Emotional deficits in schizophrenia affect diverse processes, including emotional experience (Myin-Germeys et al., 2000; Blanchard and Cohen, 2006; Horan et al., 2008; Cohen and Minor, 2010; Taylor et al., 2012), emotion expression (Blanchard and Cohen, 2006), and perception and recognition of emotional cues (Couture et al., 2006; Hoekert et al., 2007; Kohler et al., 2010; Horan et al., 2011). In contrast, other emotional skills, such as the ability to appraise the emotional valence of stimuli, appear relatively preserved in schizophrenia (Kring and Moran, 2008). However in some cases inconsistent data were collected (Strauss et al., 2011). Indeed, from one hand, recent evidences on schizophrenia impairment in emotional behavior were based upon the observation that patients displayed “blunted” or “flat” facial and vocal affect, which was supposed to reveal a correspondingly diminished emotional experience. From the other hand, available data suggests that their subjective experience of emotion is not consistently modified (Aghevli et al., 2003; Herbener et al., 2007, 2008). Further, previous studies have indicated that patient self-report of affective experience has high internal consistency (Blanchard et al., 1998; Horan et al., 2006a,b). Moreover, a number of studies have found that patients do not differ from healthy subjects in their ratings of a variety of affective stimuli with regard to valence and arousal ratings (Kring et al., 2003; Kring and Moran, 2008; Cohen and Minor, 2010). In general, results did not support a primary hedonic deficit in schizophrenia (for example, rating positive stimuli as less pleasant). Rather, data suggested that atypical emotional response is mostly associated with experiencing negative stimuli as being highly unpleasant and arousing. These potential contrasting effects can be also interpreted in terms of two broad social cognitive processes: lower-level versus higher-level (Frith and Frith, 2006; Ochsner et al., 2009; Green and Lee, 2012). Lower-level are social cognitive processes which involve recognizing significance in social and emotional stimuli (such as recognition of facial expression of emotions and vocal emotional features). These processes are considered as partially automatic neural responses to emotional cues and usually rely on specific neural circuits, including amygdala and insula. Higher-level processes involve an ability to make inferences about or contextualize representations that are driven by low-level recognition processes. These two levels may be partially preserved but they may have some difficulties to be connected, generating discordances between emotion experience, expression and recognition (Regan et al., 2015).

In the present review we mainly focused on emotional cue perception and recognition. Indeed emotion perception is one of the most relevant component of social cognition in schizophrenia and it usually involves the identification of emotions displayed in emotional patterns or in facial stimuli.

Electrophysiology (EEG) and functional Magnetic Resonance (fMRI) approaches are able to examine neural correlates for explicit as well as implicit tasks (i.e., the brain response to emotional faces during a gender identification task). It is reasonable to infer that brain imaging is appropriate for identifying various impairments in schizophrenia which are localized brain areas (Champagne et al., 2014). Nevertheless, it is limited by its low temporal resolution that does not support the real-time dynamic of the emotional filed (Logothetis, 2008). In contrast, event related potentials (ERPs), provide rate which allows milliseconds accuracy in exploring cerebral activation and a vast amount of research has already underlined its validity with emotional stimuli (Olofsson et al., 2008). Indeed, although somewhat contrasting, in general the findings from rating scales and EEG show lack of differences between schizophrenia patients (SPs) and healthy subjects: patients appear to recognize emotional stimuli to the same extent as healthy persons, even though in some cases they rate higher on anhedonia scales.

## ERPs as Measure of Schizophrenia Emotional Deficits

Event-related potential measure offers the advantage to monitor the dynamical modulations of the emotional processes, taking into account the heterogeneous levels implicated in emotional behavior. Indeed in the next paragraphs we considered ERP variation in response to emotional stimuli and facial expression patterns in SP.

### Early/Late ERPs Modulation in Emotional Tasks

Many reviews underlined the utility to apply ERP measurement in emotional study and specifically to schizophrenia (Friedman and Squires-Wheeler, 1994). Indeed it was found to be suitable to measure changes in brain activity at early and late latencies, furnishing a complete overview of the emotional processing across-time. In fact, early and late mechanisms sequentially describe the dynamic variations of subjective response to emotional contexts. This dynamic modulations are not easily accessible by classical neuroimaging measures. Specifically, some ERP deflections were explored in emotional domain, such as P100, N100, P200, P300, and late positive component (LPC; Neuhaus et al., 2010). About the early latency and middle-latency deflections (P100, N100, P200, P300), the ERP data suggest that SP emotional experience are accompanied by normal early stimulus processing and initial resource allocation processes in response to emotional stimuli (Horan et al., 2010). A consistent set of data did not evidence of hypo- or hyper-responsivity to pleasant or unpleasant stimuli in SP among these three ERP components. The topography of P100, P200, and P300 responses was also similar across SP and healthy controls, who show maximal responses in central and parietal regions of the right than left hemisphere. It was previously found that the early latency P100 is sensitive to perceptual stimulus features and indexes early sensory processing, whereas the middle-latency P200 and P300 demonstrated to be highly sensitivite to valence. The P200 response reflects early stimulus discrimination and response selection processes: it was increased in response to emotional pictures reflecting a relatively automatic attentional allocation by emotionally more arousing stimuli. The enhanced P300 amplitude to emotional stimuli is thought to reflect greater allocation of attention to emotionally relevant stimuli.

Therefore these previous results seemed to underline the quite similar profile of SP and control subjects in these early and middle- time response to emotions. However it was also found that emotionally evocative stimuli are differentially processed in SP within the first time interval of 200 ms, and, for this reason, that the early stages of emotional process may be affected (Pinheiro et al., 2013; Champagne et al., 2014). Furthermore, some recent study underlined differences in SP in later components, observing significant difference in the LPC in response to unpleasant pictures (Strauss et al., 2013). This could be due to a possible inability to downregulate emotional response by SP in a later phase of emotion processing. Specifically, about the more later potentials, a higher agreement was obtained across the studies, since the patients differed from controls in terms of valence-related amplitude and topography during the late-latency LPC interval. As in prior studies (Olofsson et al., 2008), controls showed clearly enhanced LPC amplitudes for pleasant and unpleasant as compared to neutral stimuli since LPC enhancement is believed to index sustained attentional processing of motivationally relevant stimuli (Bradley and Lang, 1994). However, the patients showed significantly less LPC differentiation between emotional (mainly pleasant) versus neutral stimuli. Hence, the pattern of results for LPC may suggest a disturbance in sustained attentional processing of pleasant stimuli in schizophrenia. In some cases LPC modulation also indicated that there was a difference in laterality between SP and control healthy subjects. Controls generally showed greater left than right hemisphere responses for pleasant stimuli whereas patients did not demonstrate any specific laterality (Dolcos and Cabeza, 2002; Cunningham and Zelazo, 2007). The left more than right asymmetry of LPC in controls is consistent with neuropsychological models that propose a significant left hemisphere specialization for pleasant emotion processing in frontal regions (Heller and Nitschke, 1998; Davidson and Irwin, 1999; Balconi et al., 2009; Balconi and Mazza, 2011; Balconi et al., 2012). Therefore these studies pointed out the significance that the lateralization effect may have to explain the emotional behavior disturbance.

It was also suggested the SP’ pattern of intact ERPs during initial processing stages but diminished differentiation between emotional versus neutral stimuli during the LPC points toward a disturbance in “affective chronometry.” This concept refers to parameters that vary over the time course of emotional behavior (Davidson, 1998). In fact, the earlier components may be represented as more stimulus-driven processes, and the later components as involving more cognitive evaluation and controlled resources. Consistent with this view, recent studies have shown that these later ERP components are regulated by top-down attentional mechanisms (Hajcak and Nieuwenhuis, 2006; Moser et al., 2006; Hajcak and Foti, 2008).

### ERP Evidences for Deficit in Emotion “Regulation” and Attention Disengagement in SP

There previous results on ERPs may support the hypothesis that SP would show an impairment in emotion regulation. Emotion regulation refers to the processes by which we modify negative and positive emotions in terms of intensity, duration, and how they are manifested. This conceptualization is based on the idea that emotions unfold as a multicomponential process, which can be regulated by using subjective strategies at different stages of emotion generation. The topic of emotion dysregulation may be explored focusing on the “negative impairment effect” found in many previous studies, which underline the anomalous response to negative patterns by SP. In fact one possible explanation for these increases in state and trait negative emotionality is that SP display impairments in “emotion regulation” (Cohen and Minor, 2010; Cohen et al., 2011; Horan et al., 2011; Strauss et al., 2011, 2013; Strauss and Gold, 2012), since cognitive change is associated with increased prefrontal cortex activity and decreased amygdala activity (Ochsner et al., 2002, 2004, 2012; Ochsner and Gross, 2005; Mocaiber et al., 2011), it is possible that the concomitant ERP findings reflect that SP either have ineffective cortical control over the amygdala and limbic regions or a failure to adequately engage prefrontal and limbic regions when applying cognitive change strategies to perform emotional task.

From another point of view, the ERP measures are usable to verify if SP had greater difficulty disengaging attention from unpleasant stimuli (Strauss et al., 2011). Indeed this impairment may play a critical role in the generation of both negative symptoms and trait negative affect. Problems with disengaging attention from unpleasant stimuli have been proposed to be a major contributor to elevated negative emotional experience in individuals with other psychiatric disorders (e.g., anxiety) (Fox et al., 2001), and SP as well (Kring and Moran, 2008; Cohen and Minor, 2010). An enhanced in negative emotion response may reflect a sustained emotional regulation problem, where patients have difficulty regulating negative mood and negative behavior (Horan et al., 2006a,b; Cohen and Minor, 2010; Cohen et al., 2011).

The present findings are consistent with prior studies demonstrating a sort of disconnection among emotional response subcomponents in SP. For example, it is well documented that SP are less expressive than healthy controls, yet do not differ as much with respect to reported emotional experience or autonomic physiology (Kring and Moran, 2008). Failure to sufficiently process motivationally relevant stimuli could have maladaptive functional consequences for SP (Dolcos and Cabeza, 2002). Diminished attentional processing reflected in the LPC could help explain why SP fail to show the memory enhancements for emotional patterns.

### Impairment in Facial Expression Comprehension

A deficit in the recognition of facial emotion is well established in SP (Walker et al., 1984; Archer et al., 1992; Schneider et al., 1995; Salem et al., 1996; Addington and Addington, 1998; Kee et al., 1998; Kohler et al., 2003; Johnston et al., 2005). This deficit appears to be at least partly related to a more general problem in cognitive functions including the categorization, discrimination, and identification of facial stimuli, and also to deficits in working memory and attentional processes (Addington and Addington, 1998; Kee et al., 1998; Kohler et al., 2000). It was revealed that anomalous late-latency activity was related to specific attentional process to facial expression, such as an attenuation of P300 response to different emotional valences (An et al., 2003). Moreover recent evidences were reported for impaired information processing in schizophrenia that occurs at the encoding level of facial stimuli. The findings in the emotion perception analysis probably reveal that SP do not modulate the amygdala to emotional versus non-emotional faces to the same extent as controls. It was also shown that SP exhibited amplitude deficits for both the late components N170, related to processing of structural components of face, and N250, related to the emotional content processing, but a latency deficit only for the N250 (Wynn et al., 2013).

## Incoming Developments: Why to Use Functional Near-Infrared Spectroscopy (fNIRS) in Schizophrenia Study on Emotions

Although the good temporal resolution of ERP enables a precise evaluation of the time-course of neural response in response to the emotion perception, its low spatial resolution makes less easy to draw accurate conclusions regarding the main neural areas involved. Therefore, the neural substrates responsible for these emotion abnormalities cannot be definitively determined using ERP alone. For this reason, recently fNIRS measure was applied to study emotions in schizophrenia (Koseki et al., 2013). fNIRS has been developed to be non-invasive, easy-to-use, portable, restraint-free, and replicable (Kono et al., 2007). Indeed fNIRS is considered to impose considerably milder physical and psychological burdens than those of classical neuroimaging techniques. It is less invasive than other techniques (for example it does not require injection of radioactive agent), and no side effects are described so far and it is thus suitable for children and patients.

Indeed fNIRS is a functional brain imaging methodology from among other available methodologies such as fMRI and positron emission tomography (PET). While both of them also have excellent spatial resolution, fMRI and PET require uneasy apparatus. In contrast, fNIRS is as portable devices which allows to use it in various and critical conditions, as studies with SP (Matsuo et al., 2003). In addition, the high temporal resolution of fNIRS is useful in characterizing the time course of prefrontal activity of psychiatric pathologies (Suto et al., 2004; Kameyama et al., 2006).

In addition, patients are examined in a normal sitting position in a quiet place without any disturbing noise and examination cost is much lower than other neuroimaging modalities. However, fNIRS also has some limitations: poor spatial resolution, inability to measure deeper cerebral structures, and the possibility of involvement of extra-cerebral structures such as the scalp, fact which makes the validity of fNIRS results to be better evaluated in the future.

Previously fNIRS has been applied to assess brain functions of patients with psychiatric disorders such as schizophrenia, bipolar disorder, depression, dementia, post-traumatic stress disorder, and pervasive developmental disorder (Fallgatter et al., 1997; Hock et al., 1997; Matsuo et al., 2004; Shinba et al., 2004; Suto et al., 2004; Kubota et al., 2005; Kameyama et al., 2006; Kuwabara et al., 2006). fNIRS was clinically applied for the assessment of psychiatric patients, reporting that the frontal lobe activation measured by fNIRS in SP during a verbal fluency task was lower than that in healthy controls (Okada et al., 1994).

Recent developments of studies on schizophrenia which used fNIRS measure has found emotional recognition impairment in SP and the multichannel fNIRS was shown to be a valid measure of this impaired functions (Shoji et al., 2013). In addition, schizophrenia has been shown to have neural network abnormalities in the social brain, which subserves social and interpersonal relationships (Mimura, 2014).

Therefore the integration between ERP and fNIRS measures may describe the main features of the emotional process, that is the dynamical evolution of such phenomenon (by both fNIRS and ERP) and the contribution by specific cortical areas in processing emotions (by fNIRS). No other integrated measures may respond so well to the nature of emotional behavior. However, due the limited number of studies which have used fNIRS actually applied to schizophrenia domain, the potentiality of this technique has to be explored in the next future.

## Conclusion

As pointed out by previous research, both ERPs and fNIRS measures may well elucidate the cortical correlates and functional features of emotional processing. Allowing a good temporal and spatial resolution, they may furnish a complete overview of SP’ deficits in emotional recognition. Moreover, their portability and easiness of application make them the favorite devices to describe the multicomponential domain of emotions.

## Conflict of Interest Statement

The authors declare that the research was conducted in the absence of any commercial or financial relationships that could be construed as a potential conflict of interest.
